# Analysis of Synonymous Codon Usage Bias of Zika Virus and Its Adaption to the Hosts

**DOI:** 10.1371/journal.pone.0166260

**Published:** 2016-11-28

**Authors:** Hongju Wang, Siqing Liu, Bo Zhang, Wenqiang Wei

**Affiliations:** 1 Medical School of Henan University, Kaifeng, China; 2 Key Laboratory of Special Pathogens and Biosafety, Center for Emerging Infectious Disease, Wuhan Institute of Virology, Chinese Academy of Sciences, Wuhan, China; Oklahoma State University, UNITED STATES

## Abstract

Zika virus (ZIKV) is a mosquito-borne virus (arbovirus) in the family *Flaviviridae*, and the symptoms caused by ZIKV infection in humans include rash, fever, arthralgia, myalgia, asthenia and conjunctivitis. Codon usage bias analysis can reveal much about the molecular evolution and host adaption of ZIKV. To gain insight into the evolutionary characteristics of ZIKV, we performed a comprehensive analysis on the codon usage pattern in 46 ZIKV strains by calculating the effective number of codons (ENc), codon adaptation index (CAI), relative synonymous codon usage (RSCU), and other indicators. The results indicate that the codon usage bias of ZIKV is relatively low. Several lines of evidence support the hypothesis that translational selection plays a role in shaping the codon usage pattern of ZIKV. The results from a correspondence analysis (CA) indicate that other factors, such as base composition, aromaticity, and hydrophobicity may also be involved in shaping the codon usage pattern of ZIKV. Additionally, the results from a comparative analysis of RSCU between ZIKV and its hosts suggest that ZIKV tends to evolve codon usage patterns that are comparable to those of its hosts. Moreover, selection pressure from *Homo sapiens* on the ZIKV RSCU patterns was found to be dominant compared with that from *Aedes aegypti* and *Aedes albopictus*. Taken together, both natural translational selection and mutation pressure are important for shaping the codon usage pattern of ZIKV. Our findings contribute to understanding the evolution of ZIKV and its adaption to its hosts.

## Introduction

Zika virus (ZIKV) is classified as a mosquito-borne arbovirus of the family *Flaviviridae*, genus *Flavivirus* [1]. This virus was first isolated from a blood sample of a Rhesus monkey in Uganda in 1947 and, before its outbreak in Oceania in 2007, it was confined to Africa and Southeast Asia [1]. Since then, ZIKV has been circulating in the Americas, and in May 2015, the first case of ZIKV originating from the Americas was reported in Brazil. Thus far, ZIKV has expanded from South America to more than 28 countries and has aroused the attention of the World Health Organization (WHO) as well as that of many governments [2, 3]. Clinical presentation of ZIKV fever is non-specific; the most common symptoms are rash, fever, arthralgia, myalgia, asthenia, and conjunctivitis. ZIKV is thought to be transmitted to humans mainly by *Aedes aegypti* and *Aedes albopictus*. The genome of ZIKV is a 10794-bp linear single strand of RNA that contains a large open-reading frame (ORF) encoding a polyprotein, which can be spliced into capsid protein (C), pro-envelope protein (prM), envelop protein (E), and seven nonstructural (NS) proteins [4]. The ZIKV genome has been detected in the blood, saliva, urine, amniotic liquid, and tissue samples [5–7]. Although ZIKV infection is often asymptomatic, symptomatic infections have also been described, and these patients usually report mild symptoms [2]. Importantly, ZIKV infection in pregnant women may lead to the fetal malformation. Specifically, ZIKV has been linked with the occurrence of microcephaly in the babies [8]. Currently, there is no effective medicine or vaccine against this virus.

All amino acids, except methionine (Met) and tryptophane (Trp), are coded by more than one synonymous codon. The alternative synonymous codons do not occur equally; they instead follow a special codon usage pattern, a phenomenon termed codon usage bias [9]. Several factors are known to contribute to codon usage bias, such as mutational bias, translational and transcriptional selection, protein structure, tRNA abundance, RNA stability, GC content, gene expression level, and gene length [10–12]. The codon usage bias is regarded as a consequence of the balance between the mutation and translational selection [13]. Analysis of codon usage bias can provide useful insights into the molecular evolution of species and their genes.

The complete genome sequencing of ZIKV has been completed [14, 15]. However, the extensive studies on the codon usage bias of ZIKV are rare. Here, we analyzed the codon usage bias of the ZIKV polyprotein-coding region and explored factors that might be related to this codon usage bias. A comprehensive analysis of ZIKV codon usage bias will be important for understanding its molecular evolution, and will provide clues for its prevention and treatment.

## Materials and methods

### Ethics Statement

This article does not contain any studies with human participants or that were performed on animals.

### Sequence data

The 46 available nucleotide sequences of ZIKV polyprotein-coding regions (Table 1) were downloaded from the NCBI GenBank database (http://www.ncbi.nlm.nih.gov/). Detailed information about these ZIKV strains is listed in Table 1.

**Table 1:** The accession number, origin and isolation year, and indices of polyprotein-coding region of each ZIKV isolate.

	Accession				GC3s^c^	GC^d^	Mononucleotide frequency [%]						GC12^k^	GC3^l^
No	number	Species	CAI^a^	ENc^b^	[%]	[%]	U3s^e^	C3s^f^	A3s^g^	G3s^h^	Gravy^i^	Aromo^j^	[%]	[%]
1	LC002520	Uganda(1947)	0.737	52.94	50.80	50.70	24.97	31.64	34.21	31.23	-0.140	0.080	49.07	53.94
2	KF383116	Senegal(1968)	0.739	52.58	51.20	51.00	24.78	31.71	33.85	31.73	-0.142	0.079	49.22	54.38
3	EU545988	Micronesia(1968)	0.735	53.64	51.80	51.00	25.28	31.93	32.65	32.28	-0.148	0.081	49.11	54.88
4	KJ776791	Polynesia(2013)	0.735	53.36	52.00	51.10	24.84	32.19	32.85	32.27	-0.147	0.081	49.11	55.08
5	KU312312	Suriname(2015)	0.735	53.40	52.00	51.10	24.79	32.24	32.86	32.24	-0.147	0.081	49.15	55.08
6	KU321639	Brazil(2015)	0.736	53.28	52.00	51.10	24.85	32.24	32.82	32.23	-0.148	0.081	49.11	55.11
7	KF383119	Senegal(2001)	0.738	52.92	50.80	50.90	25.00	31.70	34.07	31.19	-0.139	0.079	49.24	54.03
8	KF383118	Senegal(2001)	0.741	52.67	50.80	50.80	24.99	31.52	34.12	31.32	-0.151	0.078	49.14	54.03
9	KF383115	CAF ^m^(1968)	0.738	52.56	50.00	50.50	25.68	30.97	34.42	31.03	-0.140	0.080	48.98	53.13
10	KF268949	CAF(1980)	0.737	52.69	49.60	50.40	25.74	31.09	34.90	30.34	-0.142	0.080	49.08	52.87
11	KF268948	CAF(1979)	0.739	52.64	49.90	50.40	25.67	31.24	34.59	30.57	-0.141	0.080	49.07	53.17
12	KF268950	CAF(1976)	0.739	52.67	49.90	50.40	25.67	31.24	34.61	30.55	-0.140	0.080	49.07	53.14
13	KF383117	Senegal(1997)	0.735	52.77	50.30	50.60	25.66	30.72	34.03	31.68	-0.141	0.080	49.04	53.42
14	KU509998	Haiti(2014)	0.736	53.30	52.00	51.10	24.85	32.24	32.82	32.22	-0.148	0.081	49.12	55.11
15	KU501217	Guatemala(2015)	0.735	53.15	51.90	51.10	24.82	32.23	33.07	32.03	-0.144	0.081	49.11	54.91
16	KU365777	Brazil(2015)	0.735	53.27	51.90	51.10	24.83	32.22	32.95	32.14	-0.147	0.081	49.14	54.99
17	KU501216	Guatemala(2015)	0.735	53.18	51.80	51.00	24.85	32.20	33.07	32.03	-0.144	0.081	49.11	54.88
18	KU501215	PR^n^ (2015)	0.735	53.25	52.10	51.10	24.65	32.40	32.95	32.12	-0.147	0.081	49.12	55.14
19	KU365780	Brazil(2015)	0.735	53.27	51.90	51.10	24.87	32.18	32.95	32.14	-0.147	0.081	49.14	54.96
20	KU365779	Brazil(2015)	0.735	53.29	51.90	51.10	24.87	32.18	32.95	32.14	-0.147	0.081	49.12	54.96
21	KU365778	Brazil(2015)	0.735	53.34	51.90	51.10	24.83	32.22	33.03	32.05	-0.147	0.081	49.14	54.94
22	KU647676	Martinique(2015)	0.735	53.21	51.90	51.10	24.82	32.24	33.07	32.00	-0.146	0.081	49.12	54.94
23	KU866423	China(2016)	0.735	53.32	52.00	51.10	24.70	32.27	32.96	32.24	-0.147	0.081	49.14	55.11
24	KU870645	USA(2016)	0.734	53.29	51.90	51.10	24.89	32.12	32.96	32.16	-0.142	0.080	49.14	54.91
25	KU926310	Brazil(2016)	0.735	53.28	52.00	51.10	24.72	32.32	32.98	32.12	-0.147	0.081	49.12	55.05
26	KU926309	Brazil(2016)	0.736	53.22	52.10	51.20	24.67	32.31	32.86	32.30	-0.146	0.081	49.12	55.17
27	KU922960	Mexico(2016)	0.736	53.21	51.90	51.10	24.83	32.23	33.05	32.03	-0.147	0.081	49.10	54.94
28	KU820898	China(2016)	0.736	53.27	51.90	51.10	24.73	32.27	33.09	32.04	-0.146	0.081	49.14	54.96
29	KU853013	Italy(2016)	0.736	53.23	52.00	51.10	24.72	32.33	33.04	32.01	-0.146	0.081	49.17	54.99
30	KU853012	Italy(2016)	0.736	53.27	52.00	51.10	24.74	32.35	32.99	32.04	-0.147	0.081	49.15	55.02
31	KU955591	Senegal(1984)	0.740	52.67	51.20	51.00	24.91	31.58	33.73	31.91	-0.143	0.080	49.21	54.41
32	KU955592	Senegal(1984)	0.740	52.67	51.20	50.90	24.94	31.54	33.69	31.96	-0.143	0.080	49.19	54.41
33	KU955593	Cambodia(2010)	0.735	53.53	51.60	51.00	25.40	31.72	32.74	32.31	-0.148	0.081	49.17	54.73
34	KU955594	Uganda(1947)	0.738	52.89	50.90	50.80	24.79	31.80	34.20	31.25	-0.139	0.080	49.09	54.09
35	KU955595	Senegal(1984)	0.740	52.68	51.30	51.00	24.87	31.61	33.69	31.96	-0.143	0.079	49.19	54.47
36	KX056898	China(2016)	0.735	53.27	51.70	51.10	24.94	32.06	33.08	32.05	-0.147	0.081	49.17	54.82
37	KU681082	Philippines(2012)	0.735	53.55	51.50	51.00	25.29	31.69	33.04	32.07	-0.149	0.081	49.14	54.59
38	KU681081	Thailand(2014)	0.735	53.56	51.90	51.10	24.77	32.34	33.00	31.96	-0.146	0.081	49.17	55.02
39	KU761564	China(2016)	0.736	53.25	51.90	51.10	24.76	32.26	33.03	32.09	-0.146	0.081	49.14	54.99
40	KU729218	Brazil(2015)	0.735	53.33	52.00	51.10	24.93	32.09	32.81	32.31	-0.148	0.081	49.14	54.99
41	KU744693	China(2016)	0.737	53.29	52.10	51.20	24.77	32.43	32.75	32.07	-0.147	0.081	49.24	55.20
42	KU729217	Brazil(2015)	0.736	53.21	52.00	51.10	24.71	32.31	32.99	32.16	-0.146	0.081	49.15	55.02
43	KU720415	Uganda(1947)	0.738	52.91	50.90	50.70	24.86	31.75	34.21	31.23	-0.140	0.080	49.07	54.03
44	KU497555	Brazil(2015)	0.736	53.26	52.00	51.10	24.68	32.32	33.04	32.11	-0.145	0.081	49.11	55.05
45	KU707826	Brazil(2015)	0.735	53.29	51.90	51.10	24.87	32.18	32.95	32.14	-0.147	0.081	49.12	54.96
46	KU527068	Brazil(2015)	0.735	53.38	52.00	51.10	24.75	32.28	32.94	32.18	-0.144	0.081	49.13	55.05

a CAI represents codon adaptation index.

b ENc represents the effective number of codons.

c GC3s represents the frequency of the nucleotides G+C at the third positions of synonymous codons.

d GC represents the G+C content.

e U3s represents the frequency of the nucleotide U at the third positions of codons.

f C3s represent the frequency of the nucleotide C at the third positions of codons.

g A3s represents the frequency of the nucleotide A at the third positions of codons.

h G3s represents the frequency of the nucleotide G at the third positions of codons.

i Gravy represents the hydrophobicity of protein.

j Aromo represents the aromaticity of protein.

k GC12 represents the G+C content at the first and second positions of codons.

l GC3 represents the G+C content at the third positions of codons.

m represents Central African Republic.

n represents Puerto Rico.

### Phylogenetic analysis

A phylogenetic tree was drawn based on the ZIKV polyprotein-coding region using the neighbor-joining (NJ) method with a bootstrap value of 1000 replicates on MEGA6 software [16].

### Analysis of codon usage in the ZIKV polyprotein-coding region

We calculated several indicators to analyze the codon usage of the ZIKV polyprotein-coding region. The codon adaptation index (CAI) [17] was calculated by EMBOSS CAI program using human data set as a reference [18]. The relative synonymous codon usage (RSCU) [19], effective number of codons (ENc) [20], GC content, and GC3s content, hydrophobicity (GRAVY), and aromaticity (AROMO) were calculated using the CodonW 1.4.2 program (http://codonw.sourceforge.net/).

The value of relative abundance of dinucleotides (RAD) in the polyprotein-coding regions of ZIKV was calculated by computing the relevant odds ratio described by Chris Burge [21, 22]. The odds ratio $\rho_{xy}=\frac{f_{x}{}_{y}}{f_{x}\times f_{y}}$, where *f x* denotes the frequency of the nucleotide X, *f y* denotes the frequency of the nucleotide Y, and *f xy* the frequency of the dinucleotide XY. As a criterion, If *ρ*_*xy*_ >1.23 or <0.78, the XY dinucleotide is considered to be over-represented or under-represented compared with a random association of mononucleotides [22].

### Comparison between the codon usage pattern in ZIKV and those in its hosts

#### RSCU

RSCU was employed to investigate the overall synonymous codon usage bias among the genes, and this value was defined as the ratio of the observed codon usage to the expected value [23]. Codons with a RSCU value of >1.6 were regarded as over-represented, while codons with a RSCU value of <0.6 were regarded as under-represented. Codons used at an average level (no bias) have the RSCU values of 1 [24]. In our comparison of the ZIKV codon usage pattern with those of its hosts, if the RSCU value for the polyprotein-coding region of ZIKV and that of the same codon for the host were both <0.6, >1.6, or between 0.6 and 1.6, their codon usage patterns were judged to be similar [25]. The codon usage data of ZIKV’s hosts, including human (*Homo sapiens*) and mosquitoes (*A*. *aegypti* and *A*. *albopictus*) was retrieved from the codon usage database (http://www.kazusa.or.jp/codon).

#### D(A,B)

To determine the influence of the overall codon usage of hosts on that of ZIKV, the similarity index *D(A*,*B)* [26] was calculated as follows:

$$R(A,B)=\frac{\sum_{i=1}^{59}a_{i}\times b_{i}}{\sqrt{\sum_{i=1}^{59}a_{i}{}^{2}\times \sum_{i=1}^{59}b_{i}{}^{2}}}$$

$$D(A,B)=\frac{1-R(A,B)}{2}$$

where *R(A*,*B)* is termed as a cosine value of an included angle between *A* and *B* spatial vectors and represents the extent of similarity between ZIKV and hosts in the aspect of overall codon usage pattern. *a*_*i*_ is defined as the RSCU value for a specific codon among 59 synonymous codons in ZIKV polyprotein-coding region. *b*_*i*_ is defined as the RSCU value for the same codon of ZIKV’s hosts. *D(A*,*B)* represents the potential effect of the overall codon usage of the hosts on that of ZIKV, and its value varies from 0 to 1 [26]. The higher *D(A*,*B)* means the stronger influence of environment related synonymous codon usage patterns of hosts on that of ZIKV.

#### tRNA adaptation index

tRNA adaptation index (tAI) is used to estimate tRNA usage for the coding sequences of a species [27]. It represents the levels of co-adaption between a special codon and a corresponding tRNA pool and has greater correlations with protein abundance compared with other indicators [28]. The tAI value of ZIKV polyprotein-coding region based on the tRNA copy number of *H*. *sapiens* was calculated by Visual Gene Developer [29].

### Effect of mutation pressure and translational selection on the codon usage bias

#### ENc-GC3s plot

A ENc-GC3s plot was used to investigate the influence of the GC3s content on codon usage [20]. The expected ENc values for each GC3s were calculated using the following formula:

$$ENc_{\mathrm{expected}}=2+s+\frac{29}{s^{2}+{\left(1\;\text{-}\;s\right)}^{2}}$$

where s represents the GC3s value.

#### Parity rule 2 (PR2) plot

A Parity rule 2 (PR2) plot was used to assess the influence of mutation pressure and translational selection on the codon usage of genes [30]. This plot is shown by the value of AU-bias [A_3_/(A_3_+U_3_)] as the ordinate and GC-bias [G_3_/(G_3_+C_3_)] as the abscissa at the third codon position of the four-codon amino acids. The center of the plot, where both coordinates are 0.5, is the position where A = U and G = C (PR2), with no bias between influence of mutation and translational selection rates.

#### Neutrality plot (GC12 Vs GC3)

Analysis of the correlation between the GC contents at the first and second codon positions (GC12) and that at the third codon position (GC3) is useful to examine the effect of mutation pressure and translational selection on the base composition [31]. Therefore, GC12 and GC3 were calculated by using the EMBOSS CUSP program [32] and then subjected to correlation analysis.

### Correspondence analysis (CA)

Correspondence analysis (CA) is a useful multivariate statistical method for studying the internal relationship between variables and samples [33]. The mathematics procedure of CA transforms the RSCU values into a series of dimensional factors, and the results can be used to analyze the major trend in codon usage patterns among different samples. Each gene is represented with 59 dimensional variables, and each dimension matches the RSCU value of one codon, with the exclusions of AUG, UGG and stop codons. The CA was performed using the CodonW 1.4.2 program. The first two axes of CA (Axis 1 and Axis 2) were subjected to a correlation analysis.

### Statistical analysis

The statistical analyses were performed using SPSS 13.0 software package (SPSS Inc., Chicago, USA). Correlation analyses were carried out using Spearman’s rank correlation test. The *P*-values less than 0.05 were considered statistically significant.

## Results and Discussion

### Phylogenetic analysis of ZIKV based on polyprotein-coding region

**Fig 1:**
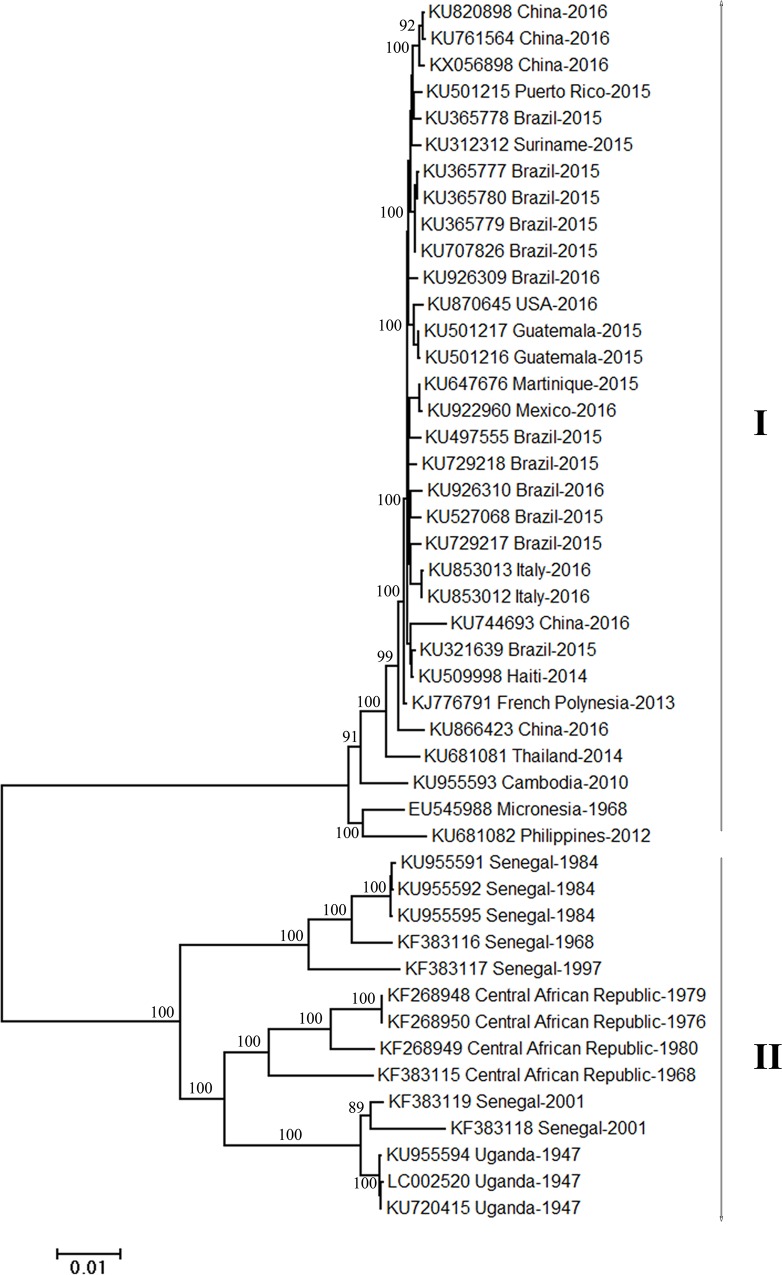
Phylogenetic tree based on the polyprotein-coding regions of 46 ZIKV strains. The tree was generated by the neighbor-joining (NJ) method using MEGA6 software. The bootstraps values were calculated with 1000 replicates.

To determine the phylogenetic relationship of different ZIKV strains, a phylogenetic tree was drawn (Fig 1). The results show that 46 strains of ZIKV can be divided into two genera (I, II) and the strains isolated from the same geographic regions cluster together (Fig 1). It can be seen that the members isolated from Africa, including Senegal, Central African Republic and Uganda, firstly cluster together and form a separate branch, and subsequently cluster with the members isolated from other countries all over the world (Fig 1).

### Nucleotide composition analysis

The GC3s content is a useful indicator of the extent of the base composition bias, representing the frequency of the nucleotides G+C at the synonymous third codon position, excluding Met, Trp, and the termination codons. The mean value of the GC contents in the 46 tested strains of ZIKV is 50.98% (50.40–51.20%; SD, 0.216), while the average value of their GC3s contents is 51.53 (49.60–52.10%; SD, 0.685) (Table 1).

An analysis of nucleotide composition at the third position of synonymous codons (G3s, A3s, U3s, C3s) indicates that the mean values of C3s (31.97%) and A3s (33.32%) are higher compared with those of G3s (31.87%) and U3s (24.95%) in ZIKV polyprotein-coding region (Table 1). Moreover, it was found that the G and A nucleotides are abundant with mean values of 29.16% and 27.50%, respectively, while the average values of U and C nucleotide were 21.52% and 21.82%, respectively (data not shown in Tables). The G and A contents are significantly higher compared with U and C contents (Student’s t test, p<0.01). These results highlight that there is a GA-rich composition in ZIKV polyprotein-coding region.

### The synonymous codon usage characteristics of the ZIKV polyprotein-coding region

ENc was used to quantify the codon usage bias of each gene [20]. ENc values can range from 20 to 61, and lower values of ENc represent higher levels of codon usage bias. To measure whether or not ZIKV strains show similar codon usage biases, the ENc values of 46 different strains were calculated. The ENc values of the ZIKV polyprotein-coding regions vary from 52.13 to 55.00, with a mean value of 53.32 and a standard deviation (SD) of 0.81, showing that the codon usage bias of ZIKV is low (Table 1).

The CAI value is a universal measure of the synonymous codon usage of genes in different organisms and can be used to analyze the adaption of a species to its hosts [17]. CAI values can range from 0 to 1 and higher CAI values signify higher levels of codon usage bias. We found that, in relation to human, the CAI values of ZIKV polyprotein-coding regions range from 0.734 to 0.741, with an average value of 0.740 and a SD of 0.002 (Table 1).

This study on 46 ZIKV strains revealed that the codon usage bias in the polyprotein-coding region of ZIKV is low as the mean ENc value of ZIKV polyprotein-coding regions is 53.32 (>40). This result is analogous to those of previous studies, which found that some RNA viruses, such as hepatitis A virus, bovine viral diarrhea virus, SARS-coronavirus, Newcastle disease virus, Marburg virus, and swine fever virus, also show a weak codon usage bias [22, 34–38]. A possible explanation for this is that the low codon usage bias may be beneficial for the efficient transcription and translation of virus genes in host cells [22]. In addition, ZIKV shows the high CAI value (0.740) for *H*. *sapiens*, suggesting that natural selection from *H*. *sapiens* can affect the codon usage of ZIKV and the evolution of codon usage in ZIKV has made it to utilize the translation resource of *H*. *sapiens* more efficiently. This is similar to Marburg virus, which also has a higher CAI value for *H*. *sapiens* but shows low codon usage bias [37].

### Relationships between the codon usage pattern of ZIKV and that of its hosts

To investigate the synonymous codon usage pattern, the RSCU values of 59 codons (excluding Met, Trp, and the termination codons) in ZIKV polyprotein-coding regions were calculated. Among 18 preferable codons, 13 have an end base of A or C, while only five have an end base of U or G; therefore, the codons with end bases of A and C are prone to be preferentially utilized in the ZIKV genome (Table 2).

**Table 2:** The relative synonymous codon usage frequency (RSCU) of ZIKV and its natural hosts.

AA^a^	Codon	RSCU^b^			
		ZIKV	*Homo sapiens*	*Aedes aegypti*	*Aedes albopictus*
Phe	UUU	1.008	0.87	0.56	0.48
	UUC	0.992	1.13	1.44	1.52
Leu	UUA	0.330	0.39	0.35	0.23
	UUG	1.323	0.73	1.34	1.11
	CUU	0.801	0.73	0.67	0.49
	CUC	1.000	1.21	0.81	0.87
	CUA	0.659	0.40	0.54	0.57
	CUG	1.892	2.53	2.28	2.73
Ile	AUU	0.864	1.03	1.00	0.74
	AUC	1.167	1.52	1.59	1.86
	AUA	0.967	0.44	0.40	0.40
Val	GUU	0.843	0.69	1.05	0.88
	GUC	1.099	1.00	1.09	1.30
	GUA	0.387	0.42	0.60	0.51
	GUG	1.668	1.90	1.26	1.31
Ser	UCU	0.854	1.11	0.67	0.54
	UCC	0.985	1.39	1.20	1.40
	UCA	1.515	0.84	0.68	0.48
	UCG	0.420	0.33	1.41	1.70
	AGU	0.997	0.84	0.93	0.79
	AGC	1.228	1.50	1.11	1.08
Pro	CCU	0.671	1.12	0.67	0.35
	CCC	1.129	1.35	0.83	1.13
	CCA	1.809	1.07	1.20	1.07
	CCG	0.391	0.46	1.30	1.44
Thr	ACU	0.980	0.94	0.80	0.64
	ACC	1.133	1.52	1.48	1.79
	ACA	1.478	1.07	0.70	0.58
	ACG	0.409	0.46	1.01	0.99
Ala	GCU	1.132	1.09	1.09	0.99
	GCC	1.290	1.64	1.48	1.81
	GCA	1.125	0.85	0.75	0.59
	GCG	0.453	0.42	0.69	0.62
Tyr	UAU	0.761	0.84	0.64	0.55
	UAC	1.239	1.16	1.36	1.45
His	CAU	0.863	0.81	0.84	0.75
	CAC	1.137	1.19	1.16	1.25
Gln	CAA	1.112	0.51	0.81	0.60
	CAG	0.888	1.49	1.19	1.40
Asn	AAU	0.692	0.89	0.79	0.64
	AAC	1.308	1.11	1.21	1.36
Lys	AAA	0.854	0.82	0.79	0.58
	AAG	1.146	1.18	1.21	1.42
Asp	GAU	0.903	0.89	1.12	0.96
	GAC	1.097	1.11	0.88	1.04
Glu	GAA	0.930	0.81	1.15	1.11
	GAG	1.070	1.19	0.85	0.89
Cys	UGU	0.941	0.86	0.83	0.69
	UGC	1.060	1.14	1.17	1.31
Arg	CGU	0.450	0.51	1.36	1.5
	CGC	0.600	1.20	1.25	1.32
	CGA	0.253	0.63	1.17	0.97
	CGG	0.559	1.20	1.05	1.22
	AGA	2.443	1.20	0.64	0.58
	AGG	1.692	1.26	0.53	0.41
Gly	GGU	0.529	0.64	1.10	1.24
	GGC	0.672	1.40	1.04	1.07
	GGA	1.793	0.98	1.49	1.21
	GGG	1.006	0.98	0.37	0.47

a AA represents amino acid.

b The “RSCU” value represents the pattern of relative synonymous codon usage.

To determine if the codon usage pattern of ZIKV is influenced by that of its hosts, the codon usage pattern of ZIKV was compared with the codon usage patterns of its natural hosts, including *H*. *sapiens*, *A*. *aegypti*, and *A*. *albopictus*. We found that 47 of 59 synonymous codons between ZIKV and *H*. *sapiens* are equivalently selected while 40 or 30 of 59 synonymous codons between ZIKV and *A*. *aegypti* or *A*. *albopictus*, respectively, are similarly selected (Table 2). In general, the similarity in the degree of codon usage between ZIKV and *H*. *sapiens* is higher than that between ZIKV and *A*. *aegypti* or *A*. *albopictus*. Specifically, CUG for leucine (Leu), AGC for serine (Ser), GCC for alanine (Ala), UAC for tryptophan (Tyr), CAC for histidine (His), AAC for asparagine (Asn), AAG for lysine (Lys), and UGC for cysteine (Cys) have high similarity between ZIKV and its natural hosts. Additionally, the RSCU values of several codons showed a strong discrepancy between ZIKV and its hosts, such as CUA for Leu, AUA for isoleucine (Ile), CCA for proline (Pro), and CGA/CGG/AGA for arginine (Arg).

These results suggest that the selection pressure from the hosts may influence the codon usage pattern of ZIKV, which may assist it in adapting to the cellular environment of the hosts and allow it to replicate efficiently in the hosts [24, 31]. Interestingly, the role of the translational selection from *H*. *sapiens* in shaping the codon usage pattern of ZIKV is different from that of its insect hosts (*A*. *aegypti* and *A*. *albopictus*). Compared with the codon usage pattern of *A*. *aegypti or A*. *albopictus*, the codon usage pattern of ZIKV is more similar to that of *H*. *sapiens*. This discrepancy of similarity in the degree of codon usage between ZIKV and its hosts may be caused by the various defense mechanisms from different hosts against ZIKV infection. Indeed, a recent study indicated that skin immune cells, including fibroblasts, epidermal keratinocytes, and immature dendritic cells, are highly permissive to ZIKV infection and replication, which can lead to the activation of an antiviral innate immune response [39]. Another study found that although *A*. *aegypti* and *A*. *albopictus* are susceptible to ZIKV infection, they are both low-competent vectors for ZIKV [40]. It is presumed that the evolution of the flavivirus genome sequence involved in anti-host countermeasures may be faster than that of other flavivirus sequence [41]. This may be one reason why the codon usage pattern of ZIKV tends to show more similarities to that of *H*. *sapiens*.

### Assessing effects of the overall codon usage of hosts on that of ZIKV

To determine how the overall codon usage of ZIKV’s hosts has contributed to virus codon usage bias, the similarity index analysis was carried out. The results indicated that all of the average values of D (*A*,*B*) among three hosts are slightly low, suggesting that ZIKV has adapted to self-replicate efficiently with strong independence of overall codon usage of its hosts during the long-term evolution. In particular, the average value of D (*A*,*B*) in *A*. *albopictus* (0.0696±0.0017) or *A*. *aegypti* (0.0528±0.0012) is higher compared with that in *H*. *sapiens* (0.0307±0.0015). This phenomenon also can be seen in the Marburg virus, in which *Rousettus aegyptiacus* exerts a more dominant effect on forming virus codon usage compared with that of *H*. *sapiens* [37].

### Relationship between dinucleotide biases and codon usage in ZIKV

Previous studies found that dinucleotide compositional constraints of genomes can affect the codon usage bias [33]. Therefore, we determined the relative abundance of 16 dinucleotides in ZIKV polyprotein-coding regions. The results show that the occurrences of dinucleotides in ZIKV are not randomly distributed and no dinucleotide is present at the expected frequency (Table 3). Specially, the dinucleotides UG and CA are over-represented (*ρ*_*xy*_ > 1.23) while UA and CG are markedly under-represented (*ρ*_*xy*_ < 0.78). These data is consistent with previous study, which suggested that the dinucleotides UA and CG are under-represented in many sequence sets [21]. Moreover, the analysis of RSCU values of the eight codons containing CG (UCG, CCG, ACG, GCG, CGU, CGC, CGA, and CGC) suggests that these codons are not preferentially used. Meanwhile, in case of UA containing codons, most of codons are not preferentially selected, except for UAC. Taken together, the composition of dinucleotides plays a role in the synonymous codon usage pattern of ZIKV.

**Table 3:** Relative abundance of the 16 dinucleotides in polyprotein-coding region of 46 ZIKV strains.

Dinucleotides	Range	Mean + SD
AA	0.944–1.004	0.987 ± 0.020
AU	0.969–1.000	0.981 ± 0.010
AC	0.904–0.984	0.967 ± 0.012
AG	1.032–1.077	1.049 ± 0.013
UA	0.514–0.553	0.535 ± 0.009
UU	0.965–1.064	0.999 ± 0.016
UC	0.985–1.036	1.015 ± 0.012
UG	1.414–1.492	1.434 ± 0.023
CA	1.286–1.357	1.308 ± 0.019
CU	1.213–1.277	1.256 ± 0.014
CC	1.099–1.148	1.112 ± 0.010
CG	0.373–0.454	0.431 ± 0.025
GA	1.049–1.159	1.123 ± 0.018
GU	0.818–0.857	0.831 ± 0.008
GC	0.990–0.992	0.931 ± 0.013
GG	0.445–1.079	1.046 ± 0.091

The relative abundance of dinucleotides has been shown to influence the codon usage in some RNA viruses [42]. In our study, we found the relative low abundances of CpG and UpA in ZIKV, which may be beneficial for the virus to escape the host anti-viral immune response and complete virus transcription reaction efficiently [43]. The unmethylated CpG can be recognized by the host innate immune system as a pathogen signature, and activates various immune response pathways [42, 44]. UpA deficiency was proposed to avail virus by reducing the risk of nonsense mutations, minimizing the improper transcription and decreasing the opportunities of cleavage by RNase L [45].

### Correspondence analysis and correlation analysis: compositional properties of the ZIKV polyprotein-coding region

The A, U, C, G, and GC contents were compared with the A3s, U3s, C3s, G3s, and GC3s contents, respectively (Table 4). The results show that correlations in nucleotide compositions are complicated. Specifically, both the G and GC contents have a significant negative correlation with the content of A3s or U3s, as well as a significant positive correlation with the content of C3s, G3s, or GC3s. The A content has a significant negative correlation with the content of C3s, G3s or GC3s and a significant positive correlation with the content of A3s or U3s. The U content has a significant negative correlation with the content of C3s or GC3s as well as a significant positive correlation with A3s or U3s content, except for the insignificant correlation between U and G3s contents. The C content has a significant negative correlation with A3s, U3s, or C3s content as well as a significant positive correlation with G3s or GC3s content. These data shows that the nucleotide compositional constraint may also affect the codon usage of ZIKV.

**Table 4:** The correlation analysis between the A, U, C, G contents and A3s, U3s, C3s, G3s contents in all selected ZIKV strains^a^.

	A3s	U3s	C3s	G3s	GC3s
**A**	0.856**	0.560**	-0.777**	-0.793**	-0.847**
**U**	0.300*	0.705**	-0.593**	-0.258	-0.579**
**C**	-0.653**	-0.764**	-0.920**	0.596**	0.882**
**G**	-0.475**	-0.376**	0.343*	0.418**	0.459**
**GC**	-0.738**	-0.725**	0.856**	0.704**	0.911**

a Value in this table is the r value of correlation analysis.

** represents *P*-value < 0.01.

* represents 0.01 < *P*-value < 0.05.

**Fig 2:**
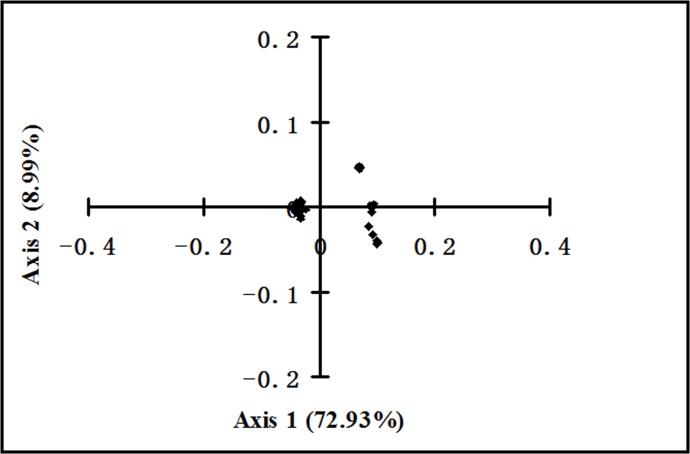
A plot of values of the first axis (Axis 1) and the second axis (Axis 2) of each polyprotein-coding region of ZIKV in correspondence analysis. The first axis accounts for 72.93% of total variation, and the second axis accounts for 8.99% of total variation.

A correspondence analysis was performed to determine the main trends in the codon usage variation and the distribution of each gene along the continuous axes. The positions of each polyprotein-coding region defined by the first axis (Axis 1) and second axis (Axis 2) are shown in Fig 2. The first axis accounts for 72.93% of the total variation, and the second, third and fourth axes account for 8.99%, 6.33%, and 3.08%, respectively, of the total variation in synonymous codon usage. A correlation analysis also showed that, except G content, the Axis1 is positively correlated with the contents of A, U, A3s, U3s, whereas it is negatively correlated with the GC3s, GC, ENc, C, C3s and G3s (Table 5). Meanwhile, Axis2 is only negatively correlated with the G content (Table 5). Overall, these results suggest that mutation pressure from the base composition plays a role in constructing the codon usage pattern of ZIKV.

**Table 5:** Correlation analysis between the first two axes and different nucleotide-relevant indices.

	GC3s	GC	ENc	A	U	C	G	A3s	U3s	C3s	G3s
**Axis1**											
*r*	-0.693**	-0.716**	-0.589**	0.748**	0.339*	-0.691**	-0.106	0.642**	0.444**	-0.624**	-0.661**
*P*	**0.000**	**0.000**	**0.000**	**0.000**	**0.021**	**0.000**	0.483	**0.000**	**0.002**	**0.000**	**0.000**
**Axis2**											
*r*	-0.072	-0.027	-0.105	0.123	-0.043	-0.053	-0.447**	0.145	0.017	0.047	-0.197
*P*	0.633	0.859	0.487	0.417	0.777	0.726	**0.002**	0.337	0.913	0.756	0.191

** represents *P*-value < 0.01.

* represents 0.01 < *P*-value < 0.05.

### The effect of translational selection on the codon usage of ZIKV

**Fig 3:**
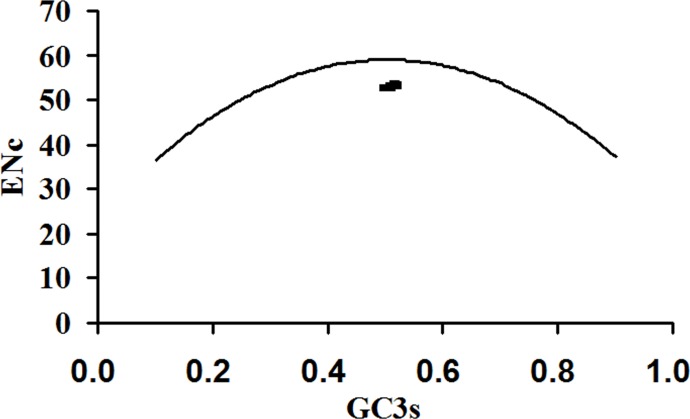
Graph showing the relationship between GC3s and effective number of codon (ENc) for the polyprotein-coding regions of ZIKV. The solid curve shows the expected ENc value if the codon usage is only determined by the variation in the GC3s.

A plot of the ENc values against the GC3s values was constructed to check the heterogeneity of codon usage [20]. If a gene is subject to the GC compositional constraints, it will lie on or near the theoretical fitting curve that represents random codon usage. In contrast, if a gene is subject to translational selection, it will lie considerably below the expected curve [46]. Here, the ENc value of each polyprotein- coding region of ZIKV was plotted against the corresponding GC3s content (Fig 3). The resulting points lie considerably below the solid curve, implying that, in addition to mutation pressure, other factors, such as translational selection, also influence the codon usage pattern of ZIKV. This result is generally similar to the related plot in previous study [34].

**Fig 4:**
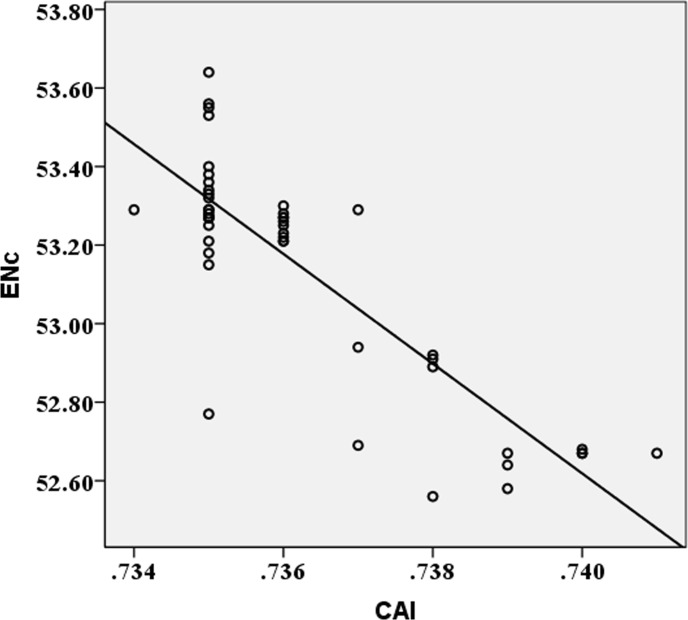
Correlation between codon adaptation index (CAI) and effective number of codons (ENc). The line represents the correlation curve produced by correlation analysis.

The base composition and codon usage bias of the ORFs of a species with an A/U-rich genome may be different from those species with G/C-rich genomes. Previous studies have employed a correlation between CAI values and ENc values to demonstrate the effect of mutation and translational selection on the codon usage bias [37, 47]. If the correlation (r) between the two indices approaches –1, this suggests that the translational selection is preferred over mutation. Otherwise, if the r value approaches 0 (no correlation), mutation may be more influential than translational selection. Our results showed that the CAI value of ZIKV is significantly positively correlated with the ENc value (r = -0.749, *P*<0.01) (Fig 4). This result reflects the influence of both translational selection and mutation pressure on the codon usage pattern of ZIKV.

**Fig 5:**
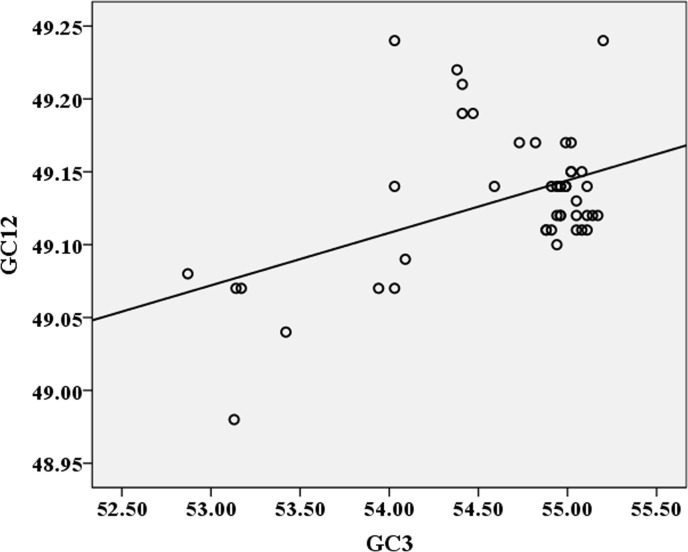
Correlation between the GC content at the first and second codon positions (GC12) and that at the third codon position (GC3). The line represents the correlation curve produced by correlation analysis.

A significant correlation between the GC12 and GC3 values is regarded to indicate that the mutation pressure dominates over the translational selection pressure in shaping the codon usage bias [22, 46]. If the correlation between GC12 and GC3 is significant, the mutation pressure is regarded as the main force forming the codon usage bias. To further determine the role of mutation pressure and translational selection in shaping the codon usage bias of ZIKV, a correlation analysis was performed to analyze the relationship between GC12 and GC3. There was no significant correlation observed between them (r = 0.25, *P*>0.05), suggesting that both translational selection and the mutation pressure are involved in shaping the codon usage pattern of ZIKV (Fig 5).

**Fig 6:**
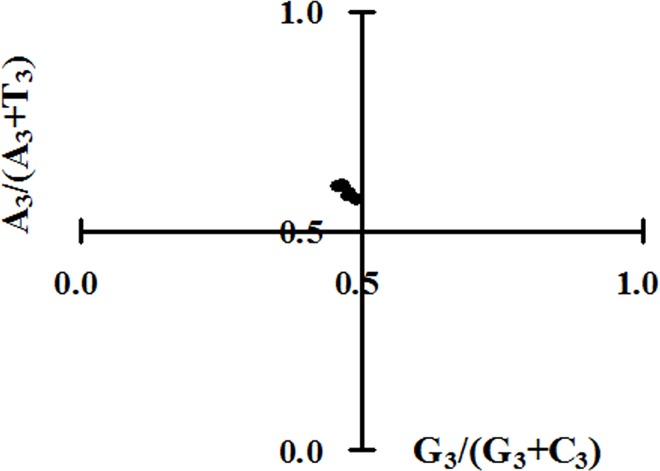
Parity rule 2 (PR2) plot [A_3_/(A_3_+U_3_) against G_3_/(G_3_+C_3_)]. PR2 bias plot was calculated for each polyprotein-coding region of ZIKV.

To determine whether the biased codon selection are restricted in highly biased coding sequences, the relationship between pyrimidines (C and U) and purines (A and G) contents in four-fold degenerate codon families (alanine, arginine, glycine, leucine, proline, serine, threonine and valine) are analyzed by PR2 bias plot. It can be seen that A and C are more frequently used than U and G in ZIKV in four-fold degenerate codon families (Fig 6). This result shows that the codon usage pattern of ZIKV is shaped by mutation pressure and other factors including translational selection.

To confirm whether translation selection from the hosts plays a role in shaping the codon usage pattern of ZIKV, the tAI values were calculated based on the tRNA copy numbers of *H*. *sapiens*. The results indicated that the tAI values of 46 ZIKV strains range from 0.329 to 0.347, with an average value of 0.344 and a SD of 0.004. Moreover, the positive correlation between tAI and CAI values (r = 0.457, *P*<0.01) in ZIKV highlights the importance of translational selection in the formation of synonymous codon usage pattern.

Compared with translational selection, mutation bias seems to have a stronger effect on the codon usage bias of some viruses [48, 49]. However, for ZIKV, the translational selection pressure also takes part in shaping the codon usage bias. Our result is consistent with a previous study that showed that recent Asian lineage spread is linked to the codon usage adaptation of the NS1 protein to human housekeeping genes [50]. During the preparation of this manuscript, two papers were published employing some ZIKV strains to analyze the codon usage [51, 52]. They concluded that mutation pressure is an important determinant of the codon usage bias of ZIKV mainly based on the result of a GC3s-ENc analysis [51]. The reasons that they do not mention the role of translational selection in the codon usage of ZIKV may be due to the lack of application of other codon usage analysis methods in their studies.

### Effect of other factors on codon usage

GRAVY and AROMO may also be related to the codon usage pattern of viruses [53]. Our correlation analysis indicated that AROMO is positively correlated with GC3s, GC, and ENc, but it is negatively correlated with Axis 1. GRAVY showed a significant positive correlation with Axis 1, but it showed a significant negative correlation with GC, GC3s, and ENc, respectively (Table 6). Both GRAVY and AROMO do not show any correlation with Axis 2. These results indicated that the aromaticity and degree of protein hydrophobicity are linked to the codon usage variation in ZIKV, emphasizing the importance of natural translational selection on forming the codon usage pattern [34].

**Table 6:** Correlation analysis among AROMO, GRAVY, the first two axes, GC3s, ENc and GC in the polyprotein-coding region of ZIKV isolates.

	Axis1	Axis2	GC3s	GC	ENc
**AROMO**					
*r*	-0.768**	-0.038	0.773**	0.787**	0.747**
*P*	**0.000**	0.803	**0.000**	**0.000**	**0.000**
**GRAVY**					
*r*	0.528**	0.101	-0.550**	-0.515**	-0.669**
*P*	**0.000**	0.506	**0.000**	**0.000**	**0.000**

** represents *P*-value < 0.01.

The involvement of aromaticity and hydrophobicity in the construction of codon usage bias has been revealed in some RNA viruses, such as bovine viral diarrhea virus, classical swine fever virus, and duck hepatitis A virus [34, 35, 54]. This study found that Axis 1 has a significant role in shaping the ZIKV codon usage pattern and is significantly correlated with aromaticity and hydrophobicity indices, implying that the aromaticity and hydrophobicity of proteins are related to the codon usage pattern of ZIKV. Aromaticity and hydrophobicity are known to play a role in peptide self-assembly and protein aggregation rates [55, 56]. A recent study showed that the structure of ZIKV particles is thermally stable, and this feature may help the virus to survive in the harsh conditions of semen, saliva, and urine [57].

**Fig 7:**
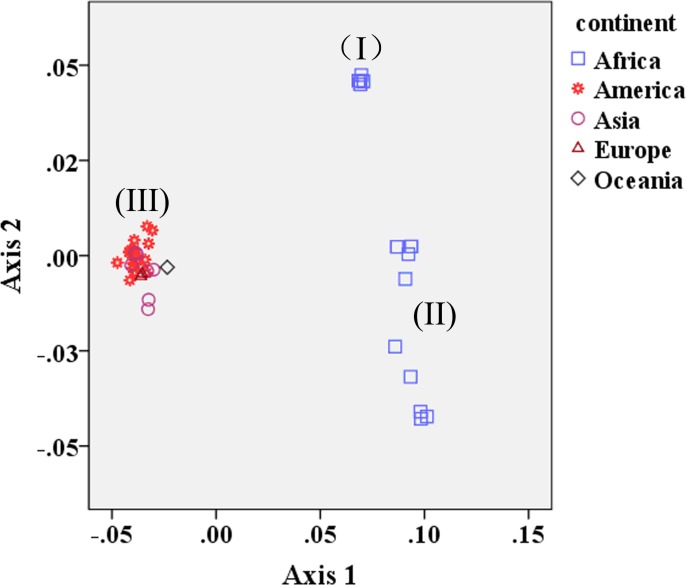
A plot of the values of the first axis (Axis 1) and the second axis (Axis 2) of ZIKV strains isolated from different continents in correspondence analysis.

It has been reported that there is a significant correlation between the phylogroups of isolates and their geographic regions, and an obvious pattern of geographic clustering has been observed in ZIKV isolates [14]. To determine if geographic factors influence the evolution of ZIKV, a plot of Axis 1 and Axis 2 was drawn according to the geographic distribution of the tested ZIKV strains. The resulting coordinate spots are separated into three groups, classified as group I, II, and III (Fig 7). Some strains isolated in Uganda clustered together with the strains isolated from Senegal, and were classified as group I. Additionally, some strains isolated from Central African Republic also clustered together with the strains isolated from Senegal, and these were classified as group II. Most of the strains isolated, regardless of their isolation countries, tended to cluster together and were classified as group III. The codon usage pattern reflects the close relationship of ZIKV strains in different geographic regions.

**Fig 8:**
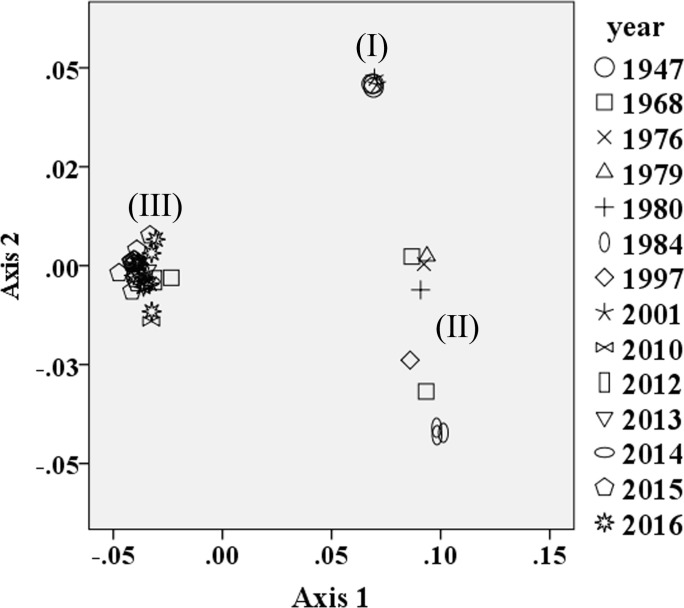
A plot of the values of the first axis (Axis 1) and the second axis (Axis 2) of ZIKV strains isolated in different years in correspondence analysis.

To investigate if the ZIKV codon usage pattern displays changes over time, a plot of Axis 1 and Axis 2 was drawn according to the outbreak time of the ZIKV strains. The 46 ZIKV isolates were divided into three groups, classified as group I, II and III (Fig 8). Most of the strains isolated from 2010 to 2016 tended to cluster together in group III. The strains isolated from 1968 to 1997 clustered together in group II, while the strains isolated in 1947 and 2001 clustered in group I. Interestingly, the strains isolated in 1968 exist in both group II and group III. These results indicated that ZIKV strains isolated in different time intervals show genetic variation in their codon usage patterns.

Previous studies showed that the Dengue virus strains occurring in the same continental region are more closely related to one another, forming a cluster when plotted by their codon usage biases, indicating that the viruses from a geographical group can show similar codon usage biases [58]. Andrew *et al* found that the geographic origin of the strains responsible for the ZIKV epidemics that occurred on Yap island in 2007 and in Cambodia in 2010 most likely originated in Southeast Asia [14]. In this study, we further found that most of the American ZIKV strains isolated in recent years cluster with some Asian, Europe and Oceania strains, supporting the idea that a close evolutionary relationship exists among Asian, Europe, Oceania and American strains.

## Conclusions

Our findings reveal that the codon usage bias of ZIKV is weak and that, in addition to mutation pressure, translational selection also influences the codon usage bias. Other factors, such as base composition, aromaticity, and hydrophobicity, also have an effect on the codon usage pattern. Importantly, there are similarities between the codon usage patterns of ZIKV and its natural hosts. This study not only provides an understanding about the variation in ZIKV codon usage patterns, but it also contributes to understanding the factors that drive ZIKV evolution.
