# Correction: Analysis of Synonymous Codon Usage Bias of Zika Virus and Its Adaption to the Hosts

**DOI:** 10.1371/journal.pone.0170128

**Published:** 2017-01-09

**Authors:** Hongju Wang, Siqing Liu, Bo Zhang, Wenqiang Wei

There is an error in Fig 6. The vertical axis is incorrectly labeled as “A_3_/(A_3_+T_3_).” The label should read “A_3_/(A_3_+U_3_).” Please view the correct Fig 6 here.

**Fig 6:**
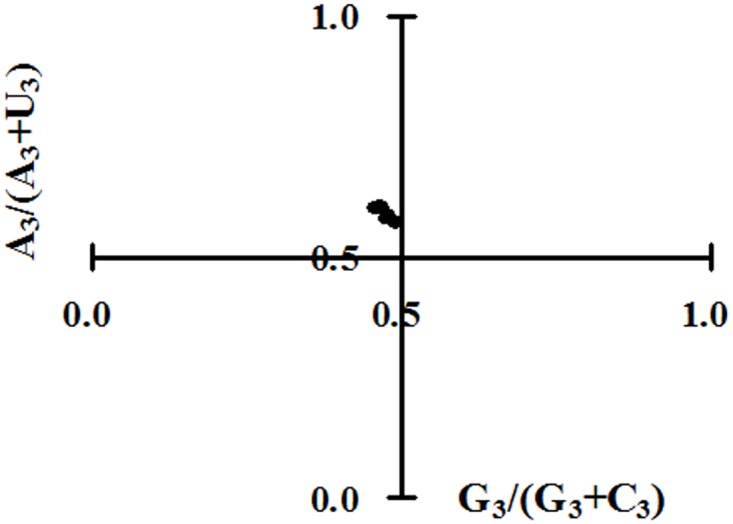
Parity rule 2 (PR2) plot [A_3_/(A_3_+U_3_) against G_3_/(G_3_+C_3_)]. PR2 bias plot was calculated for each polyprotein-coding region of ZIKV.
